# Data Set on the Use of Continuous Improvement Programs in Companies From Open-Ended Questions

**DOI:** 10.3389/fpsyg.2021.693727

**Published:** 2021-10-13

**Authors:** Amable Juarez-Tarraga, Cristina Santandreu-Mascarell, Juan A. Marin-Garcia

**Affiliations:** ^1^Departamento de Organización de Empresas, Universitat Politècnica de València, Valencia, Spain; ^2^ROGLE Departamento de Organización de Empresas, Universitat Politècnica de València, Valencia, Spain

**Keywords:** perceptions, individual suggestions systems, permanent suggestions group systems, *ad-hoc* groups, semi-autonomous groups, continuous improvement, kaizen, data paper

## Introduction

The current environment in which many industrial firms operate is characterized by intense competition with an increasingly predominant role of new technologies that develop toward smart factories, smart products and smart services embedded in an internet of things and services (Sandengen et al., 2016; Vinodh et al., 2021). In this context, the participation of workers and managers in continuous improvement programs can be a weapon for maintaining and improving competitiveness, making use of their knowledge and involvement, in order to enhancing the performance level of the entire organization (Terziovski and Sohal, 2000; Bessant et al., 2001; Van Dijk and Van Den Ende, 2002; García-Lorenzo and Prado, 2003; Wood, 2003; Lee et al., 2010; de Souza et al., 2018).

The great variety of tools, techniques, and classification criteria identified in the literature for applying participation of employees through continuous improvement practices in companies evidences its complexity (Bhuiyan and Baghel, 2005; Garcia-Sabater and Marin-Garcia, 2009; Marin-Garcia et al., 2012). For example, Lillrank et al. (2001) identified four dimensions in the design of tools in order to address the implementation of continuous improvement programs: if the activities are carried out by individuals or by groups; in the case of groups, if the groups are monofunctional or multifunctional and if they are comprised by members who are at the same level or if there are hierarchies within the group; if the activities are parallel or are integrated into the day to day life of the worker and, lastly, if the structure is permanent or is dismantled at the end of specific projects. And the Berger's (1997) classification mentions two dimensions: individual or group tasks and a parallel structure that is integrated into daily work.

These tools have been gradually introduced into companies and have been used in different ways, applying different indicators to assess their performance (Marin-Garcia, 2013; Juarez-Tarraga et al., 2016). For instance, the programs that first appeared in firms were suggestions systems., followed by quality circles and, later, improvement teams, with different configurations, were introduced (García-Lorenzo and Prado, 2003; Marin-Garcia et al., 2008).

Also, it is essential to point out that the level of use of these tools varies extensively in different working scenario depending on the organizational culture or country. According to EUROFOUND (2020) “Only one-fifth of European companies have found the secret for attaining optimal workplace well-being and business performance. ‘High investment, high involvement' workplaces have been shown to offer the best outcomes for workers and employers, boosting performance and improving job quality through increasing employee autonomy, facilitating employee involvement and promoting training and learning” and according to the results of the latest survey on European Working Conditions Survey (EWCS) (EUROFOUND, 2016) significant differences at the country level are detected for the question “Are you involved in improving the work organization or work processes of the department or organization?”.

Thus, despite their reputation and the benefits demonstrated in continuous improvement programmes, both economically (Appelbaum et al., 2000; Mathieu et al., 2008; Jaca-Garcia and Santos-Garcia, 2009; Subramony, 2009; Van Aken et al., 2010; Chalmers, 2013; Prieto and Pérez-Santana, 2014; Carnerud et al., 2018; de Souza et al., 2018; Sanchez-Ruiz and Blanco, 2019; Sánchez-Ruiz et al., 2019; Sanchez-Ruiz et al., 2020; Paipa-Galeano et al., 2020) and in terms of employee satisfaction and commitment (García et al., 2013, 2014; Jurburg et al., 2017; Stelson et al., 2017; Alvarado-Ramírez et al., 2018; Paganelli et al., 2018; Sakowski and Marcinkiewicz, 2019; Sanchez-Ruiz and Blanco, 2019; Scharf et al., 2019; Paipa-Galeano et al., 2020; Tortorella et al., 2020; Marin-Garcia and Bonavia, 2021), reports of unsuccessful application or management attempts are recurring (Easton and Jarrell, 1998; Bessant et al., 2001; Hackman and Coutu, 2009; McLean et al., 2017; Rantala et al., 2018; Sunder and Prashar, 2020; Tavana et al., 2021), and also, the effects of these initiatives on long-term benefits and their sustainability remain debated (Jaca et al., 2012; Jurburg et al., 2016; Mendez and Vila-Alonso, 2018; Gutierrez-Gutierrez and Antony, 2020).

In this context there is a need for developing studies and measurements regarding continuous improvement and its interrelationships (Bateman, 2005; Hackman and Coutu, 2009; Bonavia et al., 2015; Sanchez-Ruiz and Blanco, 2019; Marin-Garcia et al., 2020; Sanchez-Ruiz et al., 2020; Marin-Garcia and Bonavia, 2021); correctly plan the implementation of these programs, as unsuccessful implementation cause organizations to waste resources, fall short of performance objectives, rework designs, and extend time to market and by considering the right issues and the facilitators and barriers perceived by workers, enable organizations to better understand how to plan for and manage them to achieve the improvement expected, both in terms of economic performance (Hackman and Coutu, 2009; Subramony, 2009) and employee commitment and well-being (Saa Perez et al., 2001; de Koeijer et al., 2014; Mendez and Vila-Alonso, 2018).

The data set provided aims to increase understand how to effectively use this kind of programs to obtain advantages that outweigh their costs, through the responses made by workers and managers to an interview designed by authors, in which four formal participation programs are analyzed: individual suggestions systems, permanent suggestions group systems, *ad-hoc* groups and semi-autonomous groups. By means the responses and opinions of the interviewees, the data set can be analyzed from different perspectives, such us:

- Perceived benefits of the use of these practices
- Barriers and facilitators
- Differences in perceptions depending on the program implemented
- The relevance of the different contour conditions provided (country, type of company, size of company, etc.)
- Additionally, applying different perspectives, like the AMO perspective (Ability, Motivation, Opportunity) (Bailey, 1993; Marin-Garcia and Martinez Tomas, 2016) or the traditionally constructs identified by Lawler (1986) (training, communication, rewards, empowerment) the dataset can be used in order to identify facilitators and barriers for the improvement of participation programs.

And finally, potential replication studies is also available, in order to researchers can advance, extend, and deepen the processes of the implementation of participation programs for continuous improvement in companies.

## Methods

### Ethical Statement

The authors comply with the Scientific Integrity Policy and good research practices of the Universitat Politècnica de València-UPV, dated by 9/11/12. This study was reviewed and approved by Ethical Committee of the Universitat Politècnica de València-UPV (CEI_P7_18_06_19).

### Participants

A total of 1,090 employees (managers and workers) were asked about context questions and if they had participated in the following programs to promote Continuous Improvement (CI) in the past 12 months: suggestion boxes, permanent team suggestion systems, short-term team suggestion systems, and self-directed work teams. The interview questionnaire included open-ended questions to obtain data on workers' and managers' perceptions of the CI programs.

The data was obtained along seven academic courses (2008–2009 to 2014–2015) using semi-structured open-ended interviews. In order to get as many responses as possible, we chose to use Purposive snowball sampling procedure (Morse, 2009; Saunders et al., 2009; Emmel, 2013) integrating the data collection in two degrees and a MOOC taught by researchers at the UPV (Valencia, Spain), so that students conducted interviews with workers or supervisors or managers in their closest circle with the only limitation that they had to know the interviewee. Given that the students come from different countries, the sample also contains data from different countries and types of companies, although the answers are in Spanish because the interviewers are Spanish speakers.

These students previously received 40 h of training and instruction about the interview contents and the way it would be carried out. In this training, the interviewers were introduced in the concepts of relevance and accuracy, in order to ensure to having data for which the estimates are as close to the true values as possible, by minimizing biases.

By prioritizing voluntary participation through purposive snowball sampling, we have lost statistical representativeness. However, this method provides other advantages that we have valued as more important, not only at an economic level but also fundamentally for the data's reliability (Noy, 2008; Cassell et al., 2017). The interviewees have been involved in the project through the interviewers and have contributed the data voluntarily and altruistically.

### Procedure

The design of the questions and the collection of data has been carried out in three stages. In the first phase, in line with previous research conducted by our research team in this field (Conci, 2012; Marin-Garcia and Conci, 2013; Juarez-Tarraga et al., 2016), we set out to analyze in-depth the level of implementation of high involvement human resource management practices to promote continuous improvement in companies, through data and perceptions of employees of the companies. As a result of this analysis, the work focused on the four programmes identified, as they have been considered, on the basis of previous research, as the most commonly used (Lawler et al., 2001; Marin-Garcia and Bonavia, 2015; Marin-Garcia et al., 2018, 2020). The questionnaire included questions with control variables, commonly used in this type of questionnaire (Cassell et al., 2017), and the original questions that we considered of interest for our research, linked to the use of the selected practices. At this point it is important to highlight the possibilities offered by the open questions included, given that they facilitate the free expression of the opinions and perceptions of the interviewees, in order to elicit responses from respondents so that the researchers' interests do not bias the research results.

In the second phase, the identification and selection of participants was carried out as described in the previous section, with the aim of obtaining as many responses as possible.

And finally, in the third phase, the information is obtained and codified. To ensure data integrity, both the interviewers and the interviewees have carried out their tasks voluntarily. The interviews were conducted face-to-face and recorded when possible (in other cases verbatim copy of responses were written down by interviewers and checked before close the interview), the answers were anonymous, the written consent was obtained from the participants before the interview, and the participants did not receive any monetary compensation.

After conducting the interviews, the interviewers transcribe the data to a web platform to archive information.

In order to avoid errors and biases in the data transcription, the interviewers were previously trained.

The data are provided in both Excel and SPSS formats, and we have also included in this article descriptive tables (data grouping, mean values, etc.) that have been considered relevant to highlight the usefulness of the data and the possibilities of further in-depth analysis, mainly with the qualitative analysis of the data provided in the open-ended questions.

## Data Set Description

All the variables collected are linked with the implementation of the four formal participation programs that we have selected in our research: individual suggestions systems, permanent suggestions group system, *ad-hoc* groups and semi-autonomous groups. The questionnaire contains a total of 28 items, which are structured in two groups. The first part's objective is to collect data about the organization and the interviewed, and the next 18 questions are related with the formal participation programs (see Table 1).

**Table 1:** Questionnaire.

**Order**	**Part**	**Id**	**Question**	**Question in English**
1	General	V-01-01	Año	Year
2	General	V-01-02	Nombre de la localidad/pueblo y país donde trabaja el encuestado	Name of the city/town where the interviewee works
3	General	V-01-04	Actividad económica/Sector: Industrial (producción); Construcción; Servicio	Sector: □Industrial (production) □Construction □Services
4	General	V-01-05	Cantidad de trabajadores en la planta industrial, oficina, tienda o centro de trabajo donde trabaja el empleado	Number of workers in the plant
5	General	V-01-06	Tipo de empresa: Sólo una planta/oficina; Varias plantas/oficinas, en un mismo país; Varias plantas/oficinas, alguna en diferentes países	Type of company: □Only one plant/office □Several plants/offices in the same country □Several plants/offices in different countries
6	General	V-02-01	género:	V-02-01.- Sex
7	General	V-02-02	Edad en años	Age (years)
8	General	V-02-03	Años contratado en esta empresa	Years employed in this company
9	General	V-02-04	Nivel de mando: Operario (sin personas a su cargo); Mando operativo (los subordinados son operarios); Otros mandos (sus subordinados son mandos)	Management level:□Operator (without subordinates) □ Operative level (the subordinates are operators) □Other levels (the subordinates are commanders)
10	General	V-02-05	Cuántas personas trabajan en su unidad (OPERARIOS a cargo del mismo mando)	How many people work in your unit (OPERATORS under the same command)
11	Participation programs	V-03-01	¿existen Buzones de sugerencia en la empresa?	Systems of individual suggestions (suggestion boxes or similar). Do they exist in the company?
12	Participation programs	V-03-05	Si su empresa no tiene sistemas de sugerencias tipo buzón de sugerencia o similares ¿le gustaría a usted que existieran?	If your company does not have any suggestion systems, would you like them to exist?
13	Participation programs	V-03-06	¿Por qué?:	Why?
14	Participation programs	V.04.01	¿existen grupos de sugerencia permanentes en la empresa?	Suggestion systems or troubleshooting systems in PERMANENT teams (quality circles, innovation teams, Kaizen, Six Sigma). Do they exist in the company?
15	Participation programs	V-04-10	Cosas positivas que le ve a estos grupos:	Positive things you like about this system:
16	Participation programs	V-04-11	Cosas que no le gustan de estos grupos:	Things that you do not like about this system:
17	Participation programs	V-04-12	Si su empresa no tiene sistemas de sugerencias en grupos permanentes ¿le gustaría a usted que existieran/participar?:	If your company does not have any suggestion systems in permanent teams. Would you like them to exist/would you like to participate?:
18	Participation programs	V-04-13	¿Por qué?:	Why?
19	Participation programs	V-05-01	¿existen grupos *ad-hoc* en la empresa?	Suggestion systems or troubleshooting systems in SPORADIC teams (Project teams of short duration,…). Do they exist in the company?
20	Participation programs	V-05-08	Cosas positivas que le ve a este sistema	Positive things you like about this system:
21	Participation programs	V-05-09	Cosas que no le gustan de estos grupos	Things that you do not like about this system:
22	Participation programs	V-05-10	Si su empresa no tiene grupos *ad-hoc* ¿le gustaría a usted que existieran/participar?:	If your company does not have any suggestion systems in sporadic teams. Would you like them to exist/would you like to participate?:
23	Participation programs	V-05-11	¿Por qué?:	Why?
24	Participation programs	V-06-01	¿existen GRUPOS DE TRABAJO SEMIAUTÓNOMO en la empresa?	Teamwork or semi-autonomous teams. Do they exist in the company?
25	Participation programs	V-06-06	Cosas positivas que le ve a estos grupos:	Positive things you like about this system:
26	Participation programs	V-06-07	Cosas que no le gustan de Estos grupos:	Things that you do not like about this system
27	Participation programs	V-06-08	(Si su empresa no tiene GRUPOS DE TRABAJO SEMIAUTÓNOMO) ¿le gustaría a usted que existieran/participar?:	If your company does not have any teamwork or semi-autonomous teams, would you like them to exist/would you like to participate?:
28	Participation programs	V-06-09	¿Por qué?:	Why?:

Raw data are available on https://zenodo.org/record/4607445#.YFCYN9wo-Co. A descriptive analysis of these data is included in the next tables to highlight the possibility of further analysis.

We think recall bias is not likely because the questions we ask are reasonably objective and refer to four different exposures to easily identifiable CI programs. Although the responses can be affected by respondents' memory failure, this would affect the statistical power and reduce its effect on the relationship between CI and other variables, which could be higher than the results indicate (Raphael, 1987).

In Table 2, we can see descriptive information related to the closed questions:

**Table 2:**
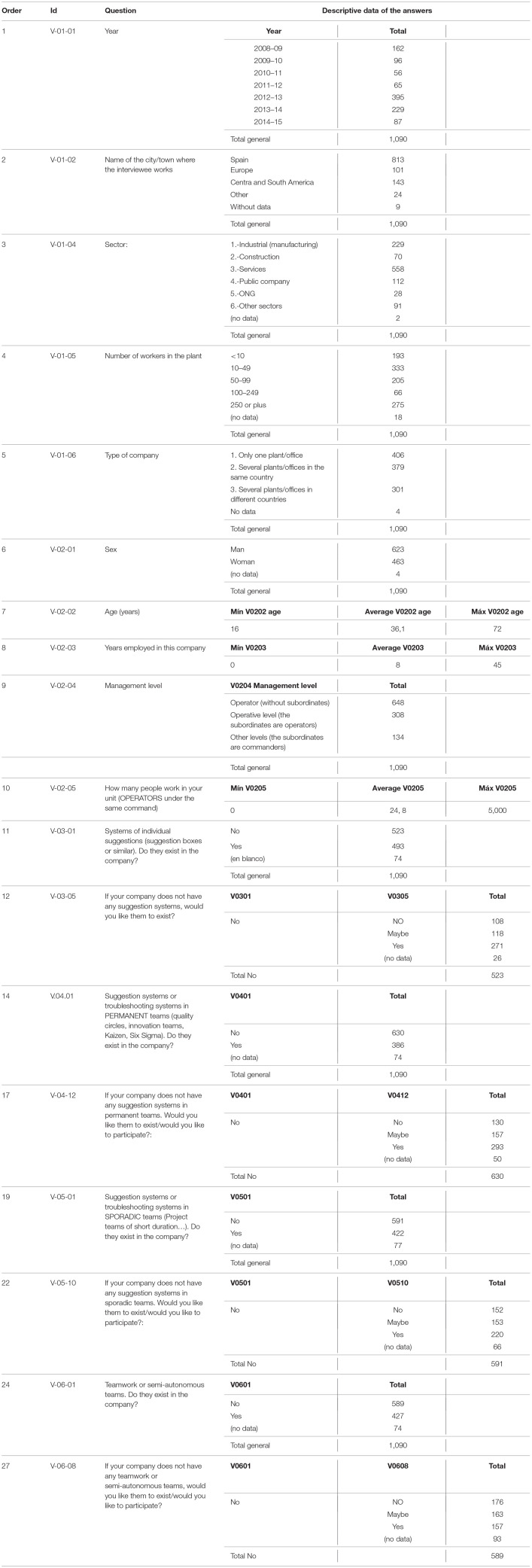
Description ended questions.

Related to the open-ended questions, in order to highlight the possibilities of a further and profound analysis of the data set, using qualitative analysis software, the future investigators can establish the coding and categorization of concepts, and the possible types of relationships/links between them, in order to generate sets of well-related concepts, linked by means of relationship statements, which together can form an integrated conceptual framework that can be used to identify or predict phenomena.

The possibilities offered by the dataset, qualitative analysis and also with mix method (quantitative and qualitative) (Fielding and Fielding, 2011; Cortini, 2014), are important, and we provide below not only the number of words available for analysis, but also the codes, categories and types of relationships that future researchers could apply:

- Numbers of words available for analysis:
  (13) V-03-06 Why?: 7,348 words
  (15) V-04-10 Positive things you like about this system: 3,205 words
  (16) V-04-11 Things that you do not like about this system: 2,098 words
  (18) V-04-13 Why?: 9,957 words
  (20) V-05-08 Positive things you like about this system: 3,253 words
  (21) V-05-09 Things that you do not like about this system: 2,106 words
  (23) V-05-11 Why?: 7,735 words
  (25) V-06-06 Positive things you like about this system: 4,249 words
  (26) V-06-07 Things that you do not like about this system: 3,221 words
  (28) V-06-09 Why?: 7,387 words
- Codes and/or categories for qualitative analysis: constraints, weakness, barriers, drawbacks, disadvantages, advantages, facilitators, strengths, and even actions (training, communication, improvement of working conditions, compensation, etc.), among others.
- Relations between codes and/or categories that can be explored: Is associated with; is part of; is cause of; contradicts; is up; is property of; difficult; no name, etc.

## Suggestions of Future Avenues of Research Using This Data Set

The data set provides information about the use of participative programs in companies and the opinions and perceptions about facilitators and barriers identified by workers and managers during these programs' implementation.

Given that the questionnaire used to obtain the data set poses some open-ended questions, in contrast to the results that can be obtained using closed questionnaires, the data set contains evoked responses that allow to obtain conclusions not predetermined by the researcher but by the interviewee (Atieno, 2009; Robinson, 2014).

The data may be of interest to researchers as well as human resources managers. Researchers on human resources and continuous improvement programs can use this data set to analyze the implementation of formal participative programs (individual suggestions systems; permanent suggestions group system; short-term improvement groups; semi-autonomous groups) and understand and investigate the team phenomena and their effectiveness. The qualitative and quantitative data obtained through the questions formulated provides a wide range of valuable information to analyze aspects as:

- Perceived benefits of the use of these practices
- Barriers and facilitators
- Implications for working conditions and employee well-being
- Actions implemented related with communication, training, compensation, participation, etc…
- Relations with the four programs analyzed and contour conditions provided (country, type of company, size of company, sector, etc.)
- Differences in perceptions depending on the program implemented
- The relevance of the different contour conditions provided (country, type of company, size of company, etc.)
- Additionally, applying different perspectives, like the AMO perspective (Ability, Motivation, Opportunity) (Bailey, 1993; Marin-Garcia and Martinez Tomas, 2016) or the traditionally constructs identified by Lawler (1986) (training, communication, rewards, empowerment) the dataset can be used in order to identify facilitators and barriers for the improvement of participation programs

Human Resource Managers interested in using these continuous improvement programs can use this data as a benchmark to know the perceptions and expectations of workers and managers.

Our data were obtained face to face in individual interviews carried out over several years, and potential replication studies are also available. Researchers can advance, extend, and deepen the processes of implementing participation programs in companies.

The complexity of the production and service provision environment present critical and new challenges for researchers and managers. Review how the approaches remain valid in companies is essential to learn if these programs (individuals and in team) are to succeed or even if synergies can be achieved (Kozlowski and Bell, 2013).

Data shared in this data article will open up doors for new research collaborations. The authors welcome future collaborations with other researchers and welcome the opportunity to contribute to a similar survey design in other countries.

## Data Availability Statement

The datasets presented in this study can be found online at: https://zenodo.org/record/4607445#.YFCYN9wo-Co.

## Ethics Statement

The authors comply with the Scientific Integrity Policy and good research practices of the Universitat Politècnica de València-UPV, dated by 9/11/12. This study was reviewed and approved by Ethical Committee of the Universitat Politècnica de València-UPV (CEI_P7_18_06_19). Written informed consent was obtained from the participants before the interview.

## Author Contributions

All authors listed have made a substantial, direct and intellectual contribution to the work, and approved it for publication.

## Conflict of Interest

The authors declare that the research was conducted in the absence of any commercial or financial relationships that could be construed as a potential conflict of interest.

## Publisher's Note

All claims expressed in this article are solely those of the authors and do not necessarily represent those of their affiliated organizations, or those of the publisher, the editors and the reviewers. Any product that may be evaluated in this article, or claim that may be made by its manufacturer, is not guaranteed or endorsed by the publisher.
